# The antigenic variability of HCV in viral HLA-Ag binding is related to the activation of the host immune response

**DOI:** 10.1038/s41598-017-15605-0

**Published:** 2017-11-14

**Authors:** P. Muñoz de Rueda, S. M. Jiménez-Ruiz, R. Quiles, E. J. Pavón-Castillero, J. A. Muñoz-Gámez, J. Casado, A. Gila, A. Ruiz-Extremera, J. Salmerón

**Affiliations:** 1 Clinical Management Unit of Digestive Diseases, Research Unit, San Cecilio University Hospital, Granada, 18012 Spain; 2 CIBER for Liver and Digestive Disease (CIBERehd), Instituto de Salud Carlos III, Madrid, 28029 Spain; 3 Instituto De Investigación Biosanitaria de Granada (ibs.GRANADA), Granada, 18012 Spain; 4 Medicine Departament, Granada University, Granada, 18016 Spain; 5 Paediatric Unit, San Cecilio University Hospital and Virgen de las Nieves University Hospital, Granada, 18012 Spain; 6 Paediatric Department, Granada University, Granada, 18016 Spain

## Abstract

Our previous data show that hepatitis C virus (HCV) genotype 1 patients expressing the HLA-DQB1 * 0301 allele have a combined response probability of 69%, while the remaining 31% do not respond, probably because the HCV immunodominant epitope (IE) against the DQB1 * 0301 allele is mutated. HCV IE (region sequenced in NS3 is a region encoding aa 1253–1272) from 37 patients (21 Sustained Virological Response, SVR; 16 non-SVR) HLA-DQB1 * 0301+, were analysed by pyrosequencing. *In vitro* cultures were also determined by CD4+ proliferation, using non-mutated IE (wild-type synthetic peptide) and synthetic mutated peptide. The pyrosequencing study revealed 34 different haplotypes. The SVR patients had fewer haplotypes (P = 0.07), mutations/haplotypes (P = 0.01) and polymorphic sites (P = 0.02) than non-SVR. Three polymorphic sites were associated with the non-SVR patients: haplotype 7 (L5P); haplotype 11 (L7P); and haplotype 15, (L15S) (P = 0.02). The *in vitro* study (n = 7) showed that in 4/7 patients (Group 1) the CD4+ proliferation obtained with wild-type synthetic peptide was higher than that obtained with the negative control and with the synthetic mutated peptide (P = 0.039). However, in the remaining 3/7 patients (Group 2) this pattern was not observed (P = 0.7). Our findings suggest that HLA-DQB1 * 0301+ patients with high antigenic variability in HCV IE (NS31253-1272) have a lower SVR rate, due to reduced CD4+ proliferation as a result of incorrect viral HLA-Ag binding.

## Introduction

Approximately 130–150 million people worldwide are infected with hepatitis C virus (HCV) and more than 500,000 die each year from liver illnesses related to this disease^1^. The mechanisms of chronicity or viral clearance depend both on the genetic characteristics of the virus and the genetic regulation of the host immune system, and the viral-specific T-cell response is believed to play a major role in determining the outcome of HCV infection. A characteristic of HCV is its high genetic variability, which can produce continual changes in its viral immunodominant epitopes (IE), thus enabling it to evade the host immune system^2–4^. Human leukocyte antigens (HLA) are responsible for the regulation and initiation of the cellular immune response. HLA class II molecules, in the connection zone with the viral antigen, present a groove that interacts with a short amino acid sequence of the antigenic peptide called the binding motif, which is located in the IE. Several IE in conserved regions of the viral genome are reported to be capable of activating CD4+ response cells. Two HLA class II alleles – HLA-DQB1 * 0301 and DRB1 * 1101 – have been associated with spontaneous viral clearance^5,6^. Specifically, HLA-DQB1 * 0301 is attached to an area of 20 amino acids located in the region of the viral genome encoding NS3 nonstructural protein. In 2011, we showed that patients with chronic hepatitis C (CHC) genotype 1 who presented the HLA-DQB1 * 0301 allele had a high rate (69%) of sustained virologic response (SVR) to pegylated interferon and ribavirin (peg-IFN + RBV) treatment^7^, although the response rates to this therapy were less than 50%^8^. We believe that when DQB1 * 0301 patients do not respond to antiviral treatment, this is partly due to the fact that HCV may escape the host immune response by generating amino acid mutations in its antigen epitopes (NS3-restricted DQB1 * 0301), thus preventing proper binding between the viral antigen and the HLA molecule.

Until 2011, the standard treatment for CHC was based on IFN-α, but in recent years, new direct-acting antivirals (DAAs), specifically targeting essential viral proteins have been introduced, in combination with IFN-based therapies and new, IFN-free regimes. The response rates to the latter treatments, over short periods of time, exceed 90%. Despite this high degree of effectiveness, numerous resistance-associated variants (RAVs) have been described in the NS3, NS5A and NS5B regions of HCV^9–14^ and more resistant virus strains may appear as these DAAs become more widely used. Hence, it is important to explore novel strategies and vaccines for treating HCV infection, in patients with mutations in viral peptide sequences, an area of crucial importance to effective antiviral treatment.

In this study, we analyse the antigenic variability of IE located in the HCV NS3 region (aa 1253–1272) in patients with CHC-1 who present the HLA DQB1 * 0301 allele, and its possible association with the effective activation of CD4+ T cells, via studies of pyrosequencing and cell cultures with synthetic peptides.

## Results

### Pyrosequencing studies

**Figure 1:**
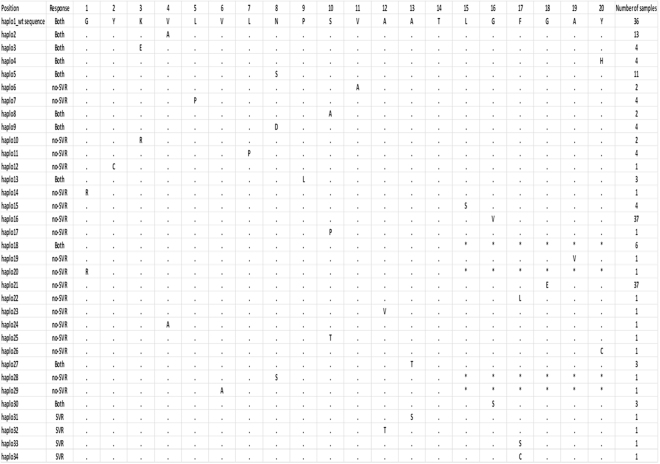
Haplotypes obtained by pyrosequencing analysis and number of samples/haplotypes. 34 different haplotypes were obtained from the pyrosequencing analysis. 29 mutated haplotypes had a single mutation; position 14 was not mutated in any haplotype; and 4 haplotypes presented a stop codon * in position 15. Of the 33 haplotypes mutated, 14 were in the patients with SVR (n = 21), and 29 in those with non-SVR (n = 16).

The sequencing of IE NS3_1253-1272_ (region sequenced in NS3 is a region encoding aa 1253–1272) was successfully performed for the 37 patients Of these, 10 were genotype 1a/ab, 7 were genotype 1a and 20 were genotype 1b. After quality control and filtering of low-quality reads (haplotypes supported by fewer than 50 reads (nucleotide sequences) were omitted, since they can be considered background noise in a sample containing thousands of reads), a total of 18197.7 pyrosequencing reads (min-max: 4815–42788) and 34 different haplotypes or genomic variants were obtained (Fig. 1). Summary reports of haplotypes (%) majority (wt) and minority for sample are shown in Supplementary Table 1 (the grey-shaded boxes indicate the absence of the haplotype). Of the 33 haplotypes mutated, 19 were specific for the non-SVR group of patients, only 4 for SVR patients and 10 for both responses (Fig. 1). Thus, in the non-SVR patients, there were 4.75 times more different mutated haplotypes than in the SVR group. On the other hand, a notable finding was that 29 mutated haplotypes had a single mutation, while four presented a stop codon at position 15, and three of these four also presented a mutation; position 14 was not mutated in any haplotype.

Table 1 summarises our findings at the amino acids sequence level, with the following information for each basal sample (n = 37): number of different haplotypes, average number of mutations per haplotype, number of polymorphic sites in the set of haplotypes, genotype and sample group: SVR and non-SVR. In the basal samples, the patients with SVR had fewer different haplotypes (P = 0.07), a lower average number of mutations per haplotypes (P = 0.01) and fewer polymorphic sites in the set of haplotypes (P = 0.02) than the non-SVR patients (Table 2).

**Table 1:** Summary report at the amino acids sequence level.

Patient	Total haplotypes	Mean number of mutations per haplotype	Polymorphic sites	Genotype	Group
P1 (Sample 33)	4	0.75	3	1a	SVR
P12 (Sample 39)	1	0	0	1a	SVR
P13 (Sample 34)	2	0.50	1	1b	SVR
P14 (Sample 29)	2	0.50	1	1b	SVR
P15 (Sample 38)	5	0.80	4	1b	SVR
P16 (Sample 37)	1	0	0	1a	SVR
P17 (Sample 47)	1	0	0	1b	SVR
P18 (Sample 40)	5	0.80	4	1a/1b	SVR
P19 (Sample 27)	1	0	0	1a/1b	SVR
P20 (Sample 31)	3	0.67	2	1b	SVR
P21 (Sample 43)	2	0.50	1	1a/1b	SVR
P22 (Sample 35)	1	0	0	1b	SVR
P23 (Sample 26)	2	0.50	1	1a	SVR
P25 (Sample 45)	2	0.50	1	1a	SVR
P28 (Sample 46)	1	0	0	1a/1b	SVR
P30 (Sample 30)	4	2.33	7	1b	SVR
P31 (Sample 48)	2	0.50	1	1a/1b	SVR
P32 (Sample 44)	2	0.50	1	1b	SVR
P33 (Sample 49)	1	0	0	1a/1b	SVR
P34 (Sample 41)	3	0.67	2	1b	SVR
P37 (Sample 50)	2	0.50	1	1b	SVR
P2 (Sample 1)	6	0.83	5	1b	Non-SVR
P5 (Sample 21)	1	0	0	1b	Non-SVR
P7 (Sample 32)	3	0.67	2	1b	Non-SVR
P26 (Sample 14)	16	0.94	12	1a	Non-SVR
P29 (Sample 16)	4	5.00	8	1b	Non-SVR
P36 (Sample 18)	1	0	0	1a	Non-SVR
P3 (Sample 6)	6	0.83	5	1b	Non-SVR
P4 (Sample 12)	4	3.50	7	1b	Non-SVR
P6 (Sample 23)	1	1.00	1	1a/1b	Non-SVR
P8 (Sample 11)	9	1.44	11	1b	Non-SVR
P9 (Sample 2)	14	0.93	11	1b	Non-SVR
P10 (Sample 15)	2	3.00	6	1b	Non-SVR
P11 (Sample 25)	1	0	0	1b	Non-SVR
P24 (Sample 20)	2	0.50	1	1a/1b	Non-SVR
P27 (Sample 24)	2	0.50	1	1a/1b	Non-SVR
P35 (Sample 22)	4	0.75	3	1a/1b	Non-SVR

**Table 2:** Statistical analysis by number of haplotypes, number of mutations/haplotype and number of polymorphic sites.

	SVR (n = 21)	non-SVR		non-SVR (n = 16)	P*	P**
		RP (n = 6)	NR (n = 10)			
Mean number of haplotypes	2.19 ± 0.2	5.17 ± 2.3	4.5 ± 1.3	4.75 ± 1.1	0.07	n.s
Mean number of mutations/haplotype	0.48 ± 0.1	1.24 ± 0.7	1.24 ± 0.8	1.24 ± 0.3	0.01	0.03
Mean number polymorphic sites	1.43 ± 0.3	4.5 ± 1.9	4.6 ± 1.3	4.56 ± 1	0.02	0.06

Sustained Virologic Response (SVR), relapse (RP), non-responder (NR). *SVR vs. non-SVR; Mann-Whitney U test. **SVR vs. RP vs. NR; Kruskal-Wallis test.

**Figure 2:**
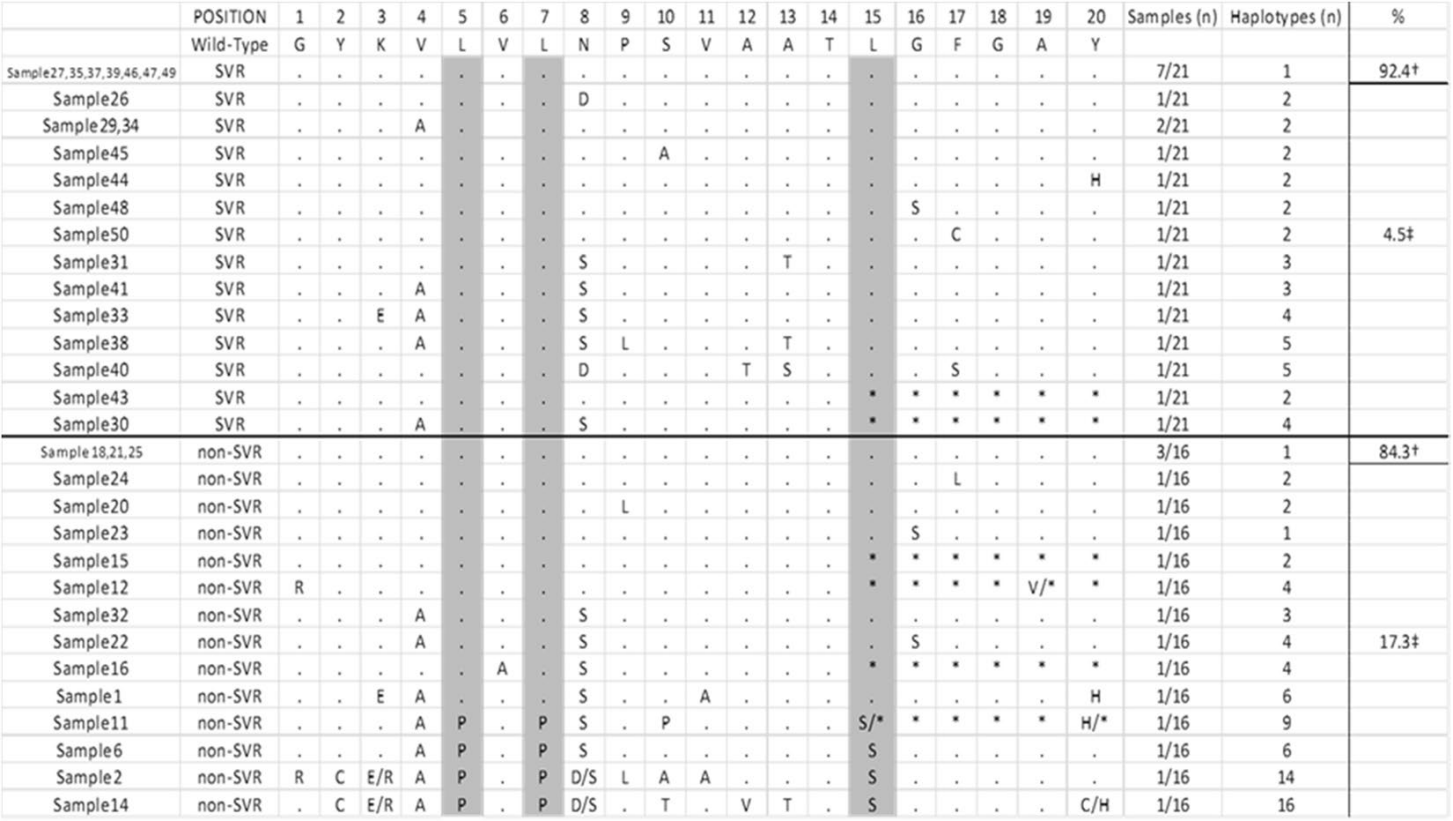
Alignment of a region covering the HLA DQB1 * 0301-restricted CD4+ T cell epitope NS3_1253-1272_. Sequences from 37 HLA DQB1 * 0301-positive patients (SVR, n = 21; non-SVR, n = 16) cohort infected with HCV genotype 1 are shown. The column % corresponds to the appearance of wild-type haplotype and mutated haplotypes. Mann-Whitney U test: ^†^P = 0.04; ^‡^P = 0.04. Positions with a significant association between the frequency of sequence polymorphisms and response are shaded in grey (Fisher’s exact test: P = 0.02).

Figure 2 shows the alignment of a region covering the HLA DQB1 * 0301-restricted CD4+ T cell epitope NS3_1253-1272_: 21 SVR and 16 non-SVR. 10/37 patients only presented wild-type haplotype (haplotype 1) (7 SVR and 3 non-SVR). The majority haplotype was the wild-type sequence, which appeared in greater proportions among the patients with SVR (SVR: 92.4% ± 3.7%; non-SVR: 84.3% ± 7.2; P = 0.04). However, the proportion of mutated haplotypes was higher among the patients with non-SVR (SVR: 4.5% ± 3.6; non-SVR: 17.3% ± 8.4; P = 0.04). Supplementary Table 1 shows that among the non-SVR patients, one (sample 23) did not present a wt haplotype and that two (samples 12 and 16) presented 4.94% and 27.53% respectively, whereas among the SVR patients, only one (sample 43) had 18.61% of the wt haplotype. Six patients had a stop codon at position 15 (2 SVR and 4 non-SVR; P > 0.05). Three polymorphic sites were associated with the non-SVR patients: haplotype 7, aa5 (L5P); haplotype 11, aa7 (L7P); and haplotype 15, aa15 (L15S) (SVR: 0/21; non-SVR: 4/16; P = 0.02; patient 3/sample 6, patient 8/sample 11, patient 9/sample 2 and patient 26/sample 14).

**Figure 3:**
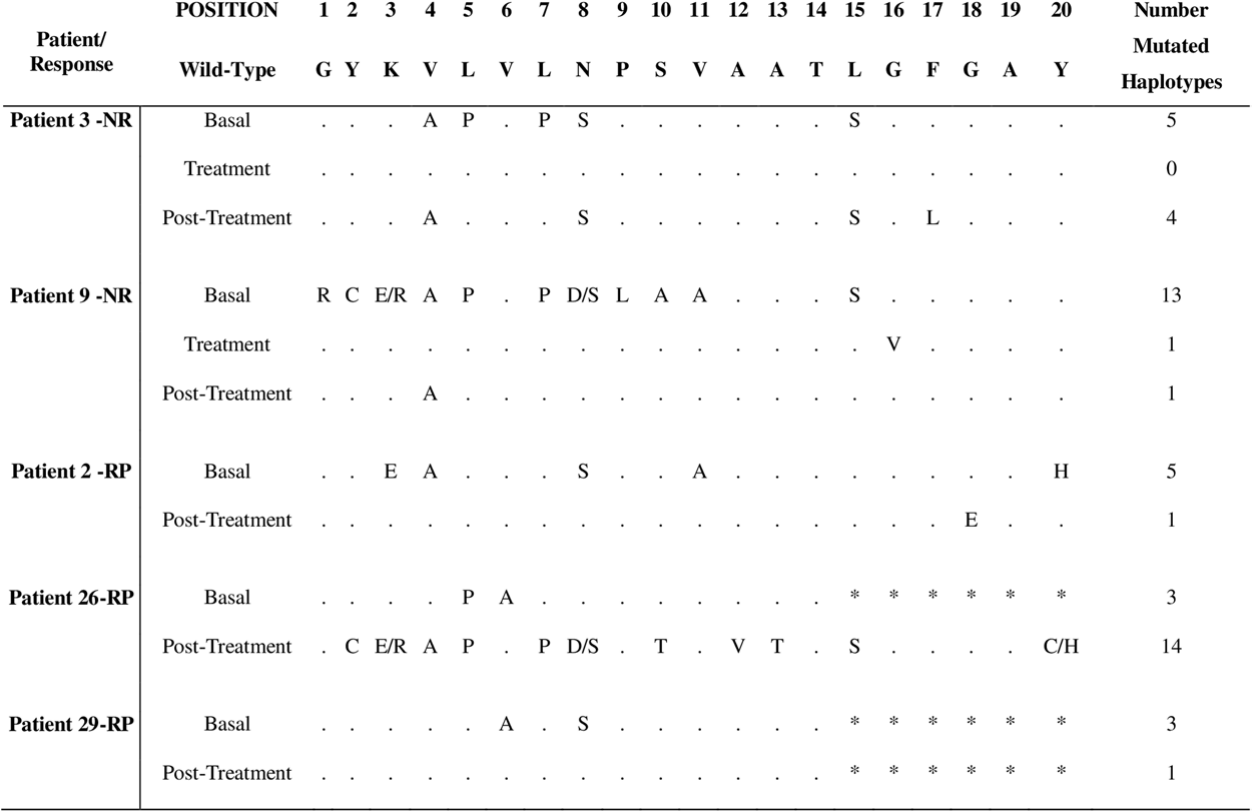
Alignment of a region covering the HLA DQB1 * 0301-restricted CD4+ T cell epitope NS3_1253-1272_ from non-SVR patients. Sequences from 5 non-SVR patients (2 patients NR and 3 patients RP) at baseline, during treatment and post-treatment are shown. No statistically significant differences were observed (P > 0.05).

Five patients with non-SVR underwent sequencing at baseline, during treatment and post-treatment. In four of them, the number of mutations had decreased by the conclusion of the treatment programme, while in the remaining patient, the number of mutations had risen (P > 0.05) (Fig. 3).

### T cell proliferative response to HCV NS3 synthetic peptides

It was unclear whether the amino acid substitutions observed in the NS3_1253-1272_ epitope restricted HLA-DQB1 * 0301 had produced an escape from the CD4+ T cell response. To address this question, we evaluated the impact of amino acid changes, individually or pooled, regarding their ability to stimulate CD4+ T cells, when isolated from the patient’s own cells. Accordingly, 7 patients were selected from the 37 who had been pyrosequenced (the inclusion criteria are described in the Method section). Of the haplotypes present in these 7 patients, the following nine synthetic peptides were designed for the *in vitro* studies and sent to Bionova for synthesis (Table 3): the wt peptide, peptides H7, H11 and H15 (corresponding to haplotypes 7, 11 and 15) which were directly associated with the non-response, and H2, H5, H30, H18 and H14 (corresponding to haplotypes 2, 5, 30, 18 and 14), which were the haplotypes found in the study patients. Each *in vitro* triplicate experiment was carried out with PBMCs from each patient, and treated with: PHA (positive control), a negative control (without stimulation), the wild-type peptide, the mutated peptides H7, H11 and H15, a pool of all three, and with each of the peptides corresponding to the haplotypes found in the viral study (pyrosequencing), together with a pool of all the latter peptides. The peptides H7, H11, H15 were used in all patients, because they were directly associated with non- response. The NS3_1253-1272_ mutated peptides (individually or pooled) were compared to the wild-type peptide in CD4+ T cell proliferation assays with PBMCs collected from each patient.

**Table 3:** Sequences of synthetic peptide variants.

Peptides	Sequence
Peptide Wild-type	GYKVLVLNPSVAATLGFGAY
Peptide H7	GYKVPVLNPSVAATLGFGAY
Peptide H11	GYKVLVPNPSVAATLGFGAY
Peptide H15	GYKVLVLNPSVAATSGFGAY
Peptide H2	GYKVLALNPSVAATLGFGAY
Peptide H5	GYKVLVLSPSVAATLGFGAY
Peptide H30	GYKVLVLNPSVAATLSFGAY
Peptide H18	GYKVLVLNPSVAAT******
Peptide H14	RYKVLVLNPSVAATLGFGAY

**Figure 4:**
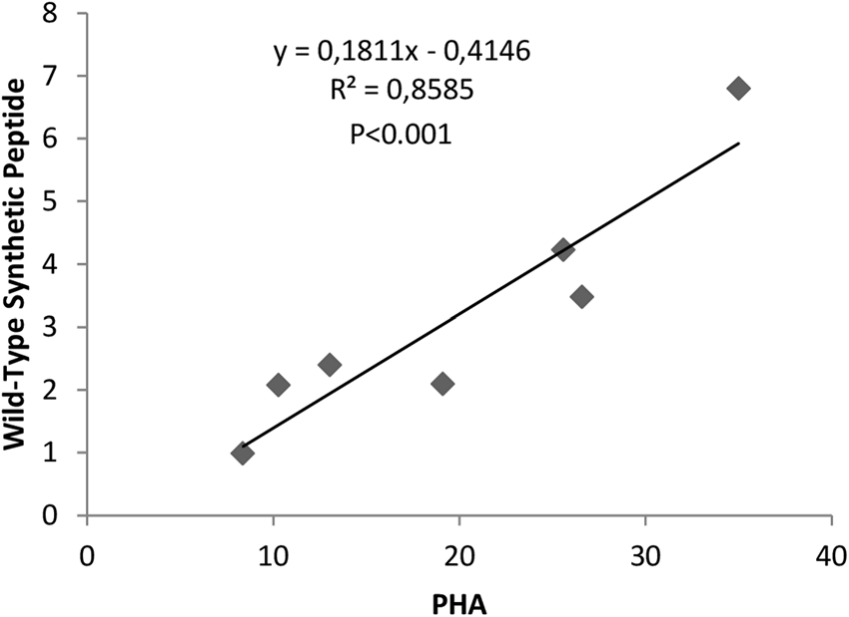
Level of stimulation of CD4+ by wild-type synthetic peptides depends on the proliferation obtained with the positive control (PHA).

A correlation study was conducted to demonstrate that the levels of stimulation of CD4+ by wild-type synthetic peptides are correlated with the proliferation obtained with the positive control. Thus, if the PHA produced little stimulation, unsurprisingly, there was little stimulation with the synthetic peptides (R2 = 0.909; P < 0.0001) (Fig. 4).

**Figure 5:**
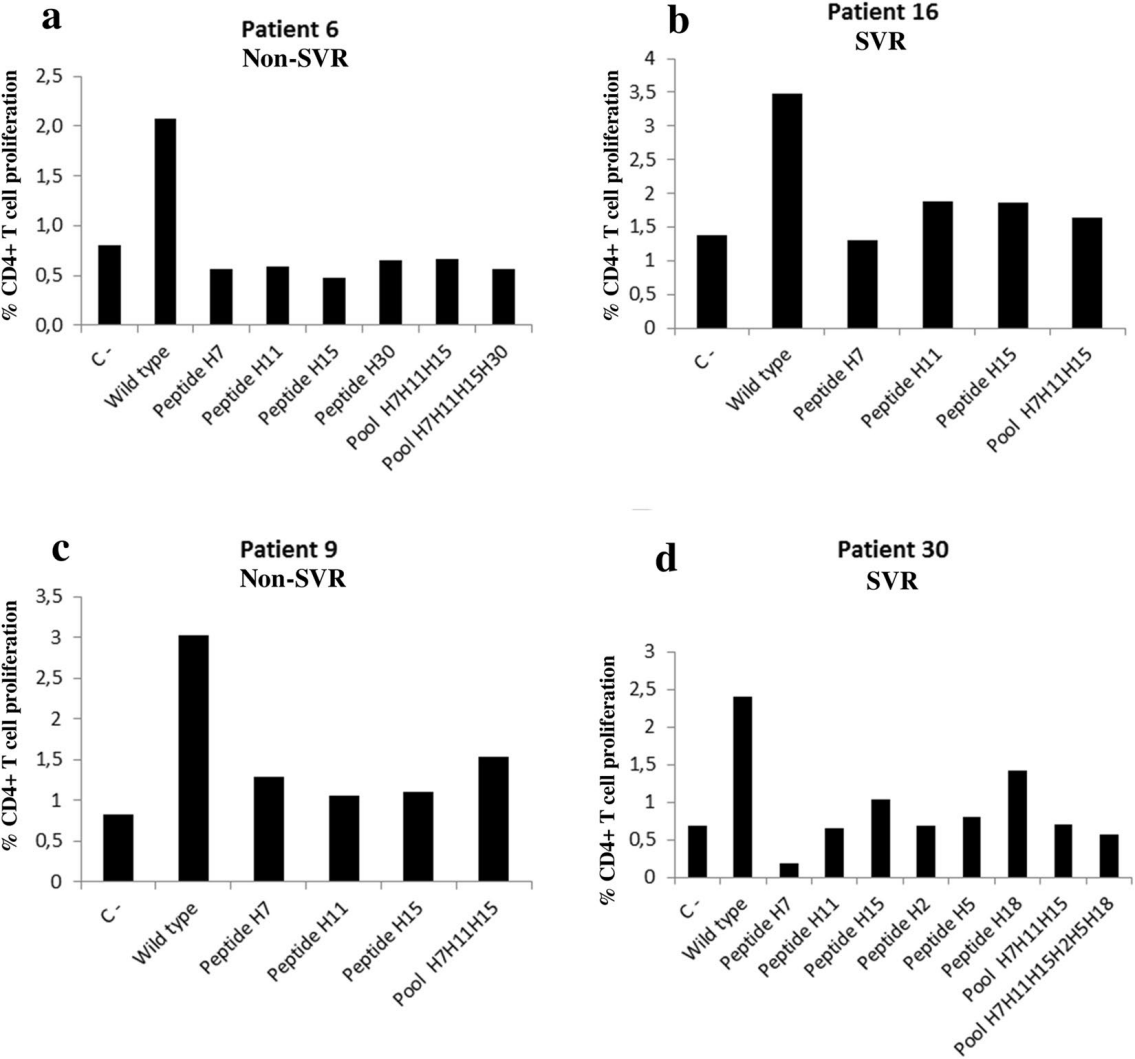
CD4+ T cell proliferation due to peptide stimulation in the Group 1 patients. In this group of patients (**a)**, patient 6; (**b**), patient 16; (**c**), patient 9 and (**d**), patient 30) the proliferation was greater due to wild-type peptide stimulation than with each of the mutated peptides (n = 4; wild peptide, 2.7% ± 0.6% vs. mutated peptide, 1.1% ± 0.5%; P = 0.039; Mann-Whitney test). C -, negative control.

In accordance with our study hypothesis, the mutated peptides did not produce an increase in proliferating CD4+ T cells, but the wild-type peptide did have this effect. Therefore, on conclusion of the study, the patients were divided into Group 1, composed of four patients among whom the hypothesis was met, and Group 2, which contained the patients who did not confirm the hypothesis. This pattern was found in four of the seven patients studied in this respect (Group 1) (n = 4; wild-type peptide, 2.7% ± 0.6% vs mutated peptides, 1.1% ± 0.5%; P = 0.039) (Fig. 5a–d).

**Figure 6:**
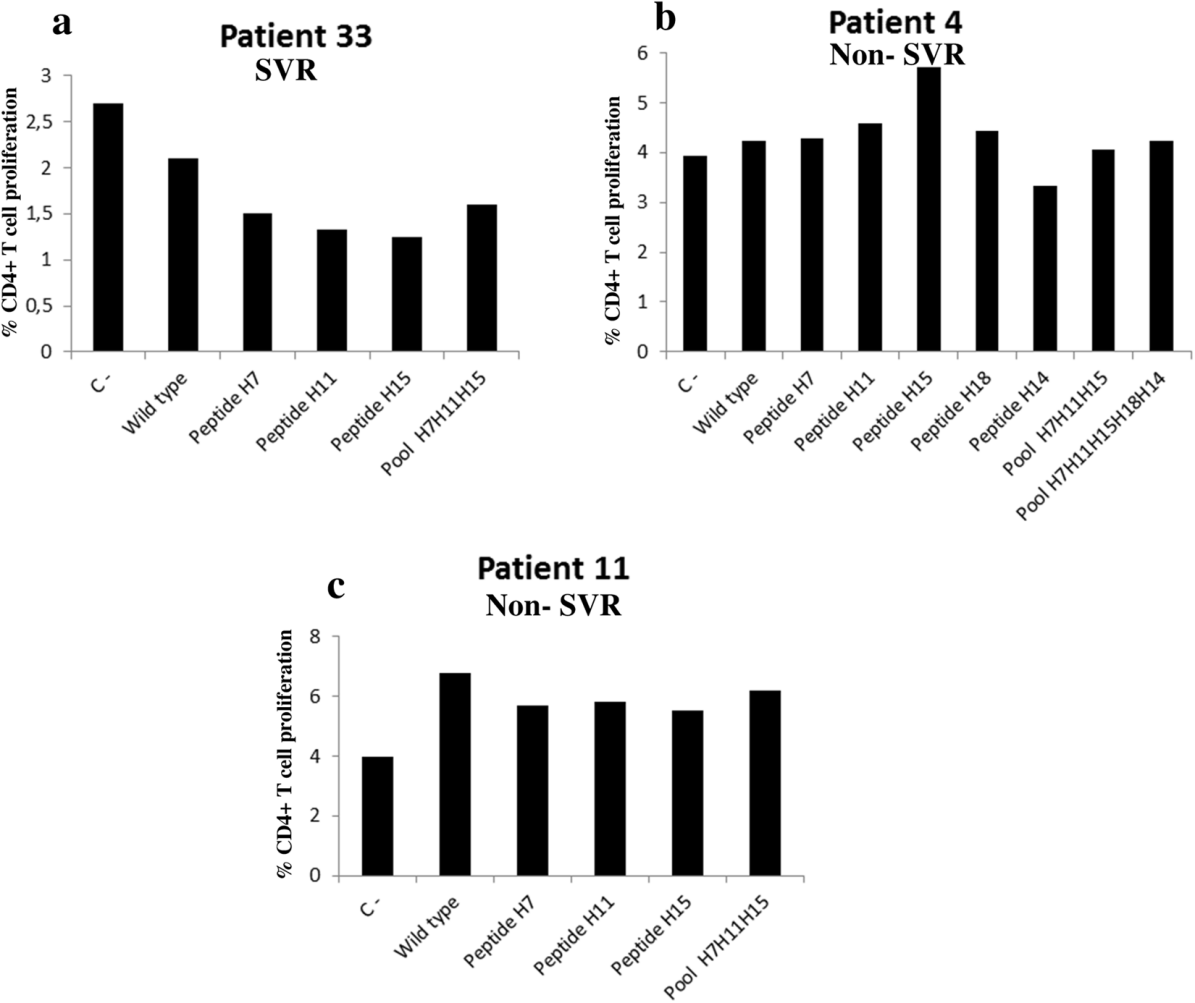
CD4+ T cell proliferation due to peptide stimulation in the Group 2 patients. In this group of patients (**a)**, patient 33; (**b**), patient 4 and (**c**), patient 11), no statistically significant differences were observed between wild-type peptide proliferation and proliferation with the mutated peptides. (n = 3; wild-type peptide, 4.3% ± 2.3% vs. mutated peptide, 4% ± 2.3%; P = 0.7; Mann-Whitney test). C −, negative control.

In the remaining three patients (Group 2) (Fig. 6a–c), this pattern was not observed and there were no statistically significant differences between the average level of proliferation with the wild-type peptide and that with all mutated peptides (n = 3; wild-type peptide, 4.3% ± 2.3% vs mutated peptide, 4% ± 2.3%; P = 0.7). The main difference observed between Group 1 and Group 2 of patients was that those who matched the expected pattern presented lower values with the negative control than did the group that did not match the pattern (negative control: 0.9% ± 0.3% vs 3.5% ± 07 respectively; P = 0.05; Mann-Whitney test), which suggests there is a lower level of immune tolerance in the patients in Group 2.

## Discussion

An effective presentation of viral antigens by HLA class I and II molecules to CD8+ and CD4+ T cells is essential for an adequate immune response to viral infection. Patients in whom the disease is chronic are known to have a lower response of CD4+ T cells^15^ than those who clear the virus^16,17^. Host genetic factors, such as HLA-DQB1 * 03^7^ and IL-28B^7,18^, have been strongly associated with spontaneous viral clearance and response to antiviral therapy. These considerations highlight the importance of the mechanism of viral HLA-Ag binding against effective CD4+ and CD8+ proliferation, in which the viral antigen, IE, plays a major role. We studied the antigenic variability of this IE, in particular NS3_1253-1272_ (by pyrosequencing) and its importance in triggering effective CD4+ activation through its HLA-DQB1 * 0301 molecule (by *in vitro* studies), finding that the presence of mutations in the IE provokes a poor response to antiviral treatment, which is partly due to the increased immune tolerance induced by a system failure antigen presentation by class II MHC molecules (DQB1 * 0301) to CD4+ T cells.

In recent years, with the advent of mass sequencing techniques it has been possible to determine the number and type of viral variants found in infected patients. By means of pyrosequencing, we can identify haplotypes that are found in very low proportions, of only 0.1% of the total population. This technique is highly sensitive^19^, unlike others commonly used in this type of study, such as direct Sanger sequencing, which has a sensitivity of only 15–20% to detect minor variants; in addition it requires extensive cloning, and is slower and more expensive^20,21^. The pyrosequencing carried out in our study shows that both the number of mutations and the number of polymorphic sites of NS3_1253–1272_ -IE are significantly greater in non-SVR patients than in SVR patients, with the non-SVR group presenting over twice as many haplotypes as the SVR group. Larrat *et al*.^22^, in a study in which pyrosequencing was carried out for the NS3 region of patients receiving triple therapy, obtained similar results, and reported that the heterogeneity of quasispecies was lower in patients with SVR than in those with non- SVR. The greater heterogeneity of quasispecies in the latter patients was also observed by Sato *et al*.^23^, who pyrosequenced the NS3 region of 34 patients at two baseline time points and at 12 hours after administering triple therapy with Telaprevir, peg-IFN and RBV. These authors found that the frequency of mutations was significantly lower at 12 hours among the patients with SVR, compared to those with non-SVR. Moreover, in the latter, the composition of viral quasispecies varied, to present resistant quasispecies.

It is not only the heterogeneity of quasispecies/haplotype that has been associated with a lower rate of SVR; this is also true of the occurrence of point mutations in the NS3 region. Thus, Dietz *et al*.^24^ found that the Q80K mutation in the NS3 region has a much higher prevalence in patients with genotype 1a than in patients with genotype 1b, generating quasispecies resistant to DAAs. In the present study, we found three polymorphic sites associated with non-SVR patients, L5P (haplotype 7); L7P (haplotype 11) and L15S (haplotype 15), and thus the three haplotypes appear simultaneously. These mutations have not been described in any previous research. In two of these mutations, there is a change of amino acid, from lysine to proline. We believe that this particular mutation is important, since the appearance of proline breaks down the α-helix in the protein structure, producing a drastic change. In our opinion, too, the change in the group of an amino acid (hydrophobic, polar, acid and basic) presupposes an alteration in the function as well as in the structure.

Numerous studies have concluded that when these IE are mutated, there is less CD4+ and CD8+ response preventing viral elimination^25–27^. In 2006, Puig *et al*.^28^ performed a study in which chimpanzees were immunised with peptides synthesised from sequences of IEs from the NS3 region, and observed that when these peptides were mutated they were not recognised by the T cells, probably due to the non-recognition of HCV by HLA class II molecules. In order to determine whether the mutations found in our patients prevented the CD4+ immune response, their own cells were cultivated against mutated synthetic peptides in the same sites corresponding to their viral population. In this case, the mutated peptides did not produce an increase in proliferating CD4+ T cells due to increased immune tolerance induced by a system failure antigen presentation by class II MHC molecules (DQB1 * 0301) to CD4+ T cells, while the wild-type peptide did cause an increase in CD4+ proliferation. Studies similar to ours have been described. Thus, Cubero *et al*.^29^ suggested that amino acid variability in the carboxy-terminal group of the NS3 domain could affect the immune response of CD4+ cells, thus contributing to the chronicity of the disease. In the latter study, cell cultures of CD4+ cells were developed and applied to a pool of 13 mutated peptides synthesised from the haplotypes found in the patient’s serum. The results obtained show that the viral population is more homogeneous when the infection is acute, and forms a bottleneck when the disease is chronic. Similar results were obtained by Wang *et al*.^30^, who reported that mutations in the epitopes of the NS3 region, in addition to producing loss of recognition of the viral antigen, can suppress the production of cytokines necessary for the immune response to occur, and are weakened by the stimulation of certain cytokines, such as IL-2.

Although our results featured considerable variation, there was, as expected, a greater stimulation of CD4+ cells by non-mutated peptides than by mutated peptides. This occurred in four of the seven patients studied (Group 1). We believe this did not occur in the other three patients (Group 2) because in these patients there was a greater proliferation of CD4+ cells receiving no stimulus, which would indicate a lower, level of immunological tolerance. This Group 2 of patients presented high baseline levels of stimulation, and therefore was not likely to achieve CD4+ proliferation with peptide stimulation.

In conclusion, although the presence of mutations does not appear to have a significant clinical impact in the new era of interferon-free DAAs^31,32^, the presence of resistant minority populations is probably relevant to the success or otherwise of therapy and the prolonged use of DAAs, since the presence of these polymorphisms may impede complete patient response, even though the response rate of the new DAAs is very high. This is why it is of great importance to continue studying the mutations of conserved regions such as NS3. Besides being a highly conserved region, this is an area in which certain IEs are required to produce an adequate immune response, and is a potential candidate for the design of synthetic vaccines. Thus, authors such as Bes *et al*.^33^ have produced the *in vitro* expansion of wild-type NS3-specific viral antigens, restoring IFN and IL-2 production to levels similar to those of responder patients.

We also believe that in the not too distant future, it will be possible to conduct a personalised follow-up of each patient, since these techniques of pyrosequencing are becoming cheaper and faster, and in this way predict the response to treatments depending on whether or not they present certain haplotypes. This knowledge can be crucial in deciding whether or not to administer treatment, this helping avoid undesirable side effects and enabling significant economic savings.

## Materials and Methods

### Pyrosequencing studies

#### Patients

The study cohort was composed of 37/100 previously-untreated CHC genotype 1 patients, presenting the HLA-DQB1 * 0301 allele, whose basal serum was frozen at 80 °C^7^. The treatment provided was combined therapy pegIFN α-2a + RBV for 48 weeks. 21 of the 37 patients achieved SVR and 16 were non-SVR. The diagnosis of CHC was based on the permanent detection of HCV-RNA serum. The patients showed no evidence of hepatitis B virus, acquired human immunodeficiency virus, alcoholism or autoimmune or drug-induced liver disease. All 37 patients underwent sequencing IE (NS3_1253-1272,_ region sequenced in NS3 is a region encoding aa 1253–1272) of HLA-DQB1 * 0301-restricted CD4+ T helper cells of HCV by pyrosequencing at baseline. Five patients with non-SVR underwent sequencing at baseline, during treatment and post-treatment. The IE sequence-restricted HLA-DQB1 * 0301 was selected from a public HCV sequence database (http://hcv.lanl.gov/; GenBank accession number AF009606).

#### Ethical Considerations

The study protocol conformed to the ethical guidelines of the 1975 Helsinki Declaration, as revised in 2013, and all patients involved in the study were informed verbally and in writing of the characteristics of the study. All these patients gave their informed signed consent to participate. This study was approved by the Ethics Committee of the San Cecilio University Hospital.

#### Hepatitis C virus NS3 RNA amplification and ultradeep pyrosequencing

Fifty samples, corresponding to 40 patients, were sent for pyrosequencing. Of these, only 44 samples from 37 patients were amplified (21 samples with SVR and 23 samples with non-SVR). The amplified HCV genome region corresponded to a fragment from gene NS3_1253–1272_. Viral RNA was extracted from 300 µL of serum using the PrepitoViral DNA/RNA 300 kit (Chemagen Technologie, PerkinElmer, Madrid, Spain). Two microlitres of the solution containing RNA were subjected to reverse transcription (RT) using a qScript Flex cDNA Synthesis Kit (Quanta Biosciences, Gaithersburg, MD). The NS3 sequences were amplified by RT-PCR using SYBR Green Supermix Low ROX (Quanta Biosciences, Gaithersburg). The primers used for RT and the first and second round amplification for the NS3 region (aa 1253−1272) are shown in Table 4. RT was performed at 42 °C for 90 min and terminated at 85 °C for 5 min, followed by the first-round PCR over 35 cycles, with each cycle consisting of denaturation at 95 °C for 30 sec, annealing at 60 °C for 30 sec and extension at 72 °C for 70 sec. The second-round PCR was performed under the same conditions. The second-round PCR product was purified using Wizard SV Gel and the PCR Clean-Up System (Promega, Promega Biotech Ibérica, Madrid, Spain) and resuspended in 20 µL of water. NS3 region (aa 1253–1272) pyrosequencing was carried out according to the manufacturer’s protocol for amplicons using the FLX 454 System (Roche SL). Bioinformatic analysis of the results obtained in the FLX 454 System (Roche SL) is presented in the Results section (Table 1).

**Table 4:** Primers used for reverse transcription and first and second-round amplification for the NS3 region (aa 1253–1272).

Application	Direction	Sequence
First PCR	Sense	5′-CAAGTGCAGCATCTACACGCGCCCACAGG-3′
RT and first PCR	Antisense	5′-TGGCACTCATCACATATTATGATGTCATAGGC-3′
Second PCR	Sense	5′-GGGAAGAGTACTAAGGTGCCGGCTGCGTATGC-3′
	Antisense	5′-CAACCACCGTCAGCTAGGAACTTGCCGTATGT-3′

### *In vitro* studies: cell cultures

#### Patients

Of the 37 patients studied by pyrosequencing, 7 (3 SVR and 4 non-SVR) were selected for the *in vitro* study. The inclusion criteria applied were that the patients should not have started a new treatment (in the case of non-SVR) and should be contactable (so that fresh blood would be available if needed). Of these 37 patients, only 7 were contacted.

#### Isolation of PBMCs

Peripheral whole blood was collected from each patient by venipuncture in lavender-top K2-EDTA BD Vacutainer tubes, to prevent clotting. Subsequently, it was aseptically mixed with saline 1:1 and the peripheral blood mononuclear cells (PBMCs) were isolated by the Ficoll Histopaque-1077 technique (Sigma Aldrich), as follows: first, 3 mL of Ficoll-1077 were introduced into a 15 mL conical tube. Then, without disturbing the interface, the mixture of whole blood and saline was trickled down the side of the tube. Subsequently, the sample was centrifuged at 800 G for 20 minutes. The tubes were carefully removed and, using a sterile Pasteur pipette, the interface where most mononuclear cells are found was transferred to a round-bottom cell culture plate. The sample was then diluted to 10 mL with PBS pH 7.4 and homogenised by inversion. After this, it was centrifuged at 100 G for 10 minutes, the supernatant was removed, the cells were rinsed with PBS (pH 7.4) and resuspended, and the previous step was then repeated. The supernatant was again removed after centrifugation and the cell pellet was resuspended with 1 mL of basic RPMI-1640 culture medium (Sigma Aldrich) supplemented with 10% foetal bovine serum (FBS) (JRH Biosciences). Cell recount was conducted in a solution of 0.4% trypan blue (Sigma Aldrich) mixed 9:1 with the cell suspension, homogenising the mixture to reach a volume of 10 µL, after which the haemocytometer count was performed. The cells were then stored in liquid nitrogen dissolved in dimethyl sulphoxide (DMSO) (Sigma Aldrich) and FBS (5:1) to maintain viability, at a cell concentration of 5 × 10^6^ mononuclear cells/mL by a stepwise procedure. For subsequent thawing, the vials were placed in a water bath at 37 °C for 2 minutes. When partially thawed, they were placed on ice and RPMI-1640 was added at 37 °C. Two washes were carried out with PBS (pH 7.4) and the cells were resuspended in 1 mL of RPMI-1640. Re-count and cell viability check were again performed, as described above.

#### Peptide synthesis

Nine synthetic peptides (NS3_1253–1272_) (Bionova) were sequenced for the *in vitro* cell culture study (Table 3). These peptides correspond to nine haplotypes found in the 7 patients included in the *in vitro* study. The peptides were HPLC purified to >98% purity. For subsequent use, they were dissolved in DMSO (Sigma Aldrich), adjusting the concentration to 1 mg/mL, and then diluting in RPMI-1640 (Sigma Aldrich) to obtain a culture dose of 8 µg/mL to stimulate the PBMCs.

#### Stimulation of PBMCs with synthetic peptides

The T cells were expanded in 15 mL round-bottom tubes at a cell concentration of 1 × 10^6^ cells/mL. Three replicates were performed for each of them, and the procedure was carried out under the same conditions for each patient, using PBMCs, previously stored in liquid nitrogen, labelled with CFSE 50 µM (Life Technologies) according to the manufacturer’s specifications, and distributed for 7 minutes at 37 °C. Once marked, the cells were washed twice with PBS to remove excess reagent, and then cultured with 400 mL of RPMI 1640 medium (Sigma Aldrich) with 25 mM HEPES, 2mM L-glutamine, 100 U/mL penicillin, 100 mg/mL streptomycin and 10% human heat-inactivated serum. Stimulation with synthetic peptides was then performed, at a peptide dose of 8 mg/mL. Phytohaemaglutinin (PHA, Sigma Aldrich) was used as a positive control, at a dose of 8 mg/mL. The cultures were incubated at 37 °C in humidified 5% CO_2_ for 5 days.

#### Flow cytometry analysis

After five days of culture, the PBMCs (at a concentration of 1 × 10^6^ cells/mL) were washed once with PBS 1% BSA and incubated with anti-CD4 PE (Beckton Dickinson) for 20 minutes at room temperature in darkness. They were then washed twice with PBS 1x + 0.5% FBS and analysed using a FACSAria Becton Dickinson II flow cytometer. Data were acquired and analysed from 1 × 10^5^ cells for every FACS analysis using Becton Dickinson 6.1 FACSDivas software (BD Biosciences, Erembodegem, Belgium).

## Electronic supplementary material

Supplementary Table 1.
