# Supplementary material for: The antigenic variability of HCV in viral HLA-Ag binding is related to the activation of the host immune response
Source: Sci Rep. 2017 Nov 14;7:15513. doi: 10.1038/s41598-017-15605-0 (PMC5686107; doi:10.1038/s41598-017-15605-0)
Supplement: Supplementary file 1 — Supplementary Table 1. [file 41598_2017_15605_MOESM1_ESM.pdf]

The antigenic variability of HCV in viral HLA-Ag binding is related to the activation of the host immune response

P. Muñoz de Rueda, S. M. Jiménez-Ruiz, R. Quiles, E. J. Pavón-Castillero, J. A. Muñoz-Gámez, J. Casado, A. Gila, A. Ruiz-Extremera, J. Salmerón

Supplementary Table 1. Summary report of haplotypes (%) majority (wt) and minority for sample (n=37).

		% Haplotype																																	
**Samples**	**Response**	**1**	**2**	**3**	**4**	**5**	**6**	**7**	**8**	**9**	**10**	**11**	**12**	**13**	**14**	**15**	**16**	**17**	**18**	**19**	**20**	**21**	**22**	**23**	**24**	**25**	**26**	**27**	**28**	**29**	**30**	**31**	**32**	**33**	**34**
**Sample1**	noRVS	94.85	.46	.31	.26	.25	.25																														
**Sample11**	noRVS	94.47	.41		.20	.27		.29				.28				.21		.32	.20																		
**Sample12**	noRVS	4.94																	85.50	4.34	1.15																
**Sample14**	noRVS	93.95	.56	.25	.21	.16		.24		.27	.20	.30	.27			.35								.49	.22	.21	.19	.17							
**Sample15**	noRVS	95.32																	.99																
**Sample16**	noRVS	27.53																	67.47										.61	.35					
**Sample18**	noRVS	97.84																																	
**Sample2**	noRVS	94.10	.42	.22		.42	.21	.34	.31	.25	.25	.24	.19	.19	.18	.17																			
**Sample20**	noRVS	93.22												3.16																					
**Sample21**	noRVS	99.72																																	
**Sample22**	noRVS	94.83	.46			.36																									.54				
**Sample23**	noRVS																														93.72				
**Sample24**	noRVS	91.78																					4.34												
**Sample25**	noRVS	94.80																																	
**Sample32**	noRVS	94.48	.34			.41																													
**Sample6**	noRVS	93.96	.39			.34		.45				.47				.42																			
**Sample26**	RVS	98.32								1.37																									
**Sample27**	RVS	99.60																																	
**Sample29**	RVS	94.93	.41																																
**Sample30**	RVS	94.60	.44																.67																
**Sample31**	RVS	94.55				.35																						.39							
**Sample33**	RVS	97.14	.36	.27		.22																													
**Sample34**	RVS	95.82	.34																																
**Sample35**	RVS	99.68																																	
**Sample37**	RVS	97.85																																	
**Sample38**	RVS	94.28	.47			.29								.40														.24							
**Sample39**	RVS	99.42																																	
**Sample40**	RVS	92.16								.33																						.75	.49	.39	
**Sample41**	RVS	94.56	.34			1.12																													
**Sample43**	RVS	18.61																	78.06																
**Sample44**	RVS	86.41			9.02																														
**Sample45**	RVS	99.16							.26																										
**Sample46**	RVS	99.81																																	
**Sample47**	RVS	99.08																																	
**Sample48**	RVS	90.54																													3.31				
**Sample49**	RVS	97.32																																	
**Sample50**	RVS	96.67																																	1.95
